# The complete mitochondrial genome of *Takydromus septentrionalis* (Reptilia: Lacertidae)

**DOI:** 10.1080/23802359.2019.1623123

**Published:** 2019-07-10

**Authors:** Jian-Guo Hu, Li-Fang Peng, Xin-Sheng Tang, Song Huang

**Affiliations:** a School of Nursing, Anhui Sanlian University, Hefei, China;; b College of Life and Environment Sciences, Huangshan University, Huangshan, China

**Keywords:** Mitogenome, *Takydromus septentrionalis*, phylogeny

## Abstract

The complete mitochondrial genome sequence of *Takydromus septentrionalis* was determined by shotgun sequencing. The total length of mitogenome is 18,304 bp, and contains 13 protein-coding genes, 22 tRNA genes, 2 ribosome RNA genes, and 2 control regions. Most of the genes of *T*. *septentrionalis* were distributed on the H-strand, except for the ND6 subunit gene and eight tRNA genes which were encoded on the L-strand. The phylogenetic tree of *T*. *septentrionalis* and 8 other closely related species was reconstructed. The phylogenetic analyses based on these mitogenomes presented here will be useful for further insights on the evolutionary relationships of *Takydromus*.

The genus *Takydromus Daudin*, 1802 contains 22 known species (Arnold et al. 2007; Uetz and Hallermann 2019). Takydromus septentrionalis Günther, 1864 was described by Günther (1864). This species is a diurnal lizards and currently widely distributed in most of China (Zhao et al. 1999; Lu et al. 2000; Huang 2002; Han et al. 2007). In this research, we determined and described the mitogenome sequence of *T. septentrionalis* in order to obtain basic genetic information about this species.

The specimen of *T*. *septentrionalis* was collected from Jiulongfeng Nature Reserve, Huangshan, Anhui, China on May 28, 2017. It was preserved and deposited in the Museum of Huangshan University (Voucher numbers: HS17112). Total genomic DNA was extracted from liver using a Qiagin DNEasy blood and tissue extraction kit (Qiagen Inc., Valencia, CA, USA). The complete mitogenome sequence has been submitted to GenBank with accession number is MK630237.

The complete mitochondrial genome sequence of *T*. *septentrionalis* has been obtained from shotgun sequencing. The total length of the complete mitogenome of *T*. *septentrionalis* was sequenced to be 18,304 bp which consisted of 13 typical vertebrate protein-coding genes (PCGs), 22 transfer RNA (tRNA) genes, 2 ribosomal RNA (rRNA) genes, and 2 control regions (D-loop). The base composition was 31.6% for A, 30.3% for T, 13.2% for G and 24.9% for C. The positions of RNA genes were predicted by the MITOS (Bernt et al. 2013), and the locations of protein-coding genes were identified by comparing with the homologous genes of other related species. Most of the *T*. *septentrionalis* mitochondrial genes are encoded on the H-strand except for the ND6 gene and eight tRNA genes, which are encoded on the L-strand. Among the mitochondrial protein-coding genes, the ATP8 was the shortest, while the ND5 was the longest. The gene order, contents and base composition are identical to those found in typical vertebrates (Boore 1999; Sorenson et al. 1999).

The phylogenetic tree of *T*. *septentrionalis* was constructed based on the complete mtDNA sequences with other 8 related species from GenBank by MEGA 7.0 (Kumar et al. 2016) using Maximum-likelihood (ML) methods. The ML tree (Figure 1) was reconstructed in http://www.phylo.org/portal2/login!input.action. As shown in Figure 1, the *T*. *septentrionalis* was close to *T*. *wolteri*. The phylogenetic analysis result was consistent with the previous research with a high support. It indicated that our new determined mitogenome sequences could meet the demands and explain some evolution issues.

**Figure 1.**
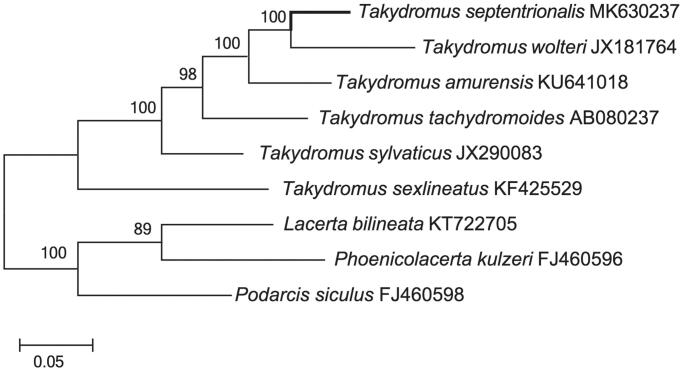
A maximum likelihood (ML) tree of *T. septentrionalis* in this study and 8 related species was constructed based on the dataset of the whole mitochondrial genome by online tool RAxML. The numbers above the branch meant bootstrap value. Bold black branches highlighted the study species and corresponding phylogenetic classification.

## References

[CIT0001] Arnold EN, Arribas O, Carranza S 2007 Systematics of the Palaearctic and Oriental lizard tribe Lacertini (Squamata: Lacertidae: Lacertinae), with descriptions of eight new genera. Zootaxa. 1430:1–86.

[CIT0002] Bernt M, Donath A, Juhling F, Externbrink F, Florentz C, Fritzsch G, Putz J, Middendorf M, Stadler PF 2013 MITOS: improved de novo metazoan mitochondrial genome annotation. Mol Phylogenet Evol. 69:313–319. 22982435 10.1016/j.ympev.2012.08.023

[CIT0003] Boore JL 1999 Animal mitochondrial genomes. Nucleic Acids Res. 27:1767–1780. 10101183 10.1093/nar/27.8.1767 PMC148383

[CIT0004] Günther A 1864 The reptiles of British India. London: Taylor & Francis; p. xxvii + 452.

[CIT0005] Han JG, Ma HQ, Wang HJ, et al. 2007 A new record of *Takydromus septentrionalis* in Hebei province, China. Chin J Zool. 42:113–113.

[CIT0006] Huang HY 2002 A New Record of *Takydromus septentrionalis* in Guangdong province, China. Sichuan J Zool. 21:37–37.

[CIT0012] Kumar S, Stecher G, Tamura K. 2016 MEGA7: Molecular Evolutionary Genetics Analysis version 7.0 for bigger datasets. Molecular Biology and Evolution. 33:1870–1874. 27004904 10.1093/molbev/msw054 PMC8210823

[CIT0008] Lu YY, Zhang P, Wang XA, et al. 2000 A New Record of *Takydromus septentrionalis* in Shandong province, China. Sichuan J Zool. 19:155–155.

[CIT0009] Sorenson MD, Ast JC, Dimcheff DE, Yuri T, Mindell DP 1999 Primers for a PCR-based approach to mitochondrial genome sequencing in birds and other vertebrates. Mol Phylogenet Evol. 12:105–114. 10381314 10.1006/mpev.1998.0602

[CIT0010] Uetz P, Hallermann J 2019. August 2018 Takydromus 1 August 2018. The Reptile Database.

[CIT0011] Zhao EM, Zhao KT, Zhou KY, et al. 1999 Fauna Sinica. Reptilia. Vol. 2 Beijing: Science Press.

